# Quantitative ^13^C MultiCP solid-state NMR as a tool for evaluation of cellulose crystallinity index measured directly inside sugarcane biomass

**DOI:** 10.1186/s13068-015-0292-1

**Published:** 2015-08-05

**Authors:** Oigres Daniel Bernardinelli, Marisa Aparecida Lima, Camila Alves Rezende, Igor Polikarpov, Eduardo Ribeiro deAzevedo

**Affiliations:** Instituto de Física de São Carlos, Universidade de São Paulo, Caixa Postal 369, São Carlos, SP 13660-970 Brazil; Instituto de Química, Universidade de Campinas-UNICAMP, Caixa Postal 6154, Campinas, SP 13084-971 Brazil

**Keywords:** Sugarcane bagasse, Biomass, Crystallinity index, Solid-state NMR, Pretreatments, Cellulosic ethanol

## Abstract

**Background:**

The crystallinity index (CI) is often associated with changes in cellulose structure after biological and physicochemical pretreatments. While some results obtained with lignocellulosic biomass demonstrate a progressive increase in the CI as a function of pretreatments, it is also shown that the CI can significantly vary depending on the choice of the measurement method. Besides, the influence of the CI on the recalcitrance of biomass has been controversial for a long time, but the most recent results tend to point out that the efficiency of pretreatments in reducing the recalcitrance is not clearly correlated with the decrease of the CI. Much of this controversy is somewhat associated with the inability to distinguish between the CI of the cellulose inside the biomass and the CI of the full biomass, which contains other amorphous components such as lignin and hemicellulose.

**Results:**

Cross polarization by multiple contact periods (Multi-CP) method was used to obtain quantitative ^13^C solid-state nuclear magnetic resonance (ssNMR) spectra of sugarcane bagasse biomass submitted to two-step pretreatments and/or enzymatic hydrolysis. By comparing the dipolar filtered Multi-CP ^13^C NMR spectra of untreated bagasse samples with those of samples submitted to acid pretreatment, we show that a 1% H_2_SO_4_-assisted pretreatment was very effective in removing practically all the hemicellulose signals. This led us to propose a spectral editing procedure based on the subtraction of MultiCP spectra of acid-treated biomass from that of the extracted lignin, to obtain a virtually pure cellulose spectrum. Based on this idea, we were able to evaluate the CI of the native cellulose inside the sugarcane bagasse biomass.

**Conclusions:**

The results show the validity of the proposed method as a tool for evaluating the variations in the CI of the cellulose inside biomasses of similar kinds. Despite a clear increase in the CI of biomass as measured by X-ray diffraction, no significant variations were observed in the CI of the cellulose inside the biomass after a particular 1% H_2_SO_4_/0.25–4% NaOH chemical-assisted pretreatments. The CI of cellulose inside the biomass solid fraction that remained after the enzymatic hydrolysis was also evaluated. The results show a slight increase in crystallinity.

## Background

The use of bio-ethanol for fuel production has increased worldwide due to environmental and economical concerns. Ethanol is now used in a number of countries to reduce automotive pollutant emissions, alleviate dependence on imported oil and also decrease risks of interruption of domestic oil production [1]. Although ethanol is currently obtained from sugarcane or corn by fermentation, this process is still not fully efficient due to the generation of large amounts of waste, for instance, the bagasse resulting from sugarcane milling. Currently, about 25–30 tons of bagasse are produced per 100 tons of milled sugarcane or 10 tons of sugar [2]. The ligno(hemi)cellulosic matrix of the biomass contains a significant amount of energy, which could be released and used. For example, sugarcane bagasse left after juice extraction can be burned to supply the steam demand of the sugar mill, and extra amounts of steam can be exported for electric power generation. Therefore, future biorefineries that process the whole sugarcane plant and other lignocellulosic biomasses will be able to produce liquid fuels, sugar, electricity and possibly a large variety of chemical products [3].

Plant biomass is mostly made up of cellulose (35–50%), followed by hemicellulose (20–35%) and lignin (10–25%). Ash, proteins and extractives make up the remaining fractions [1, 3, 4]. Cellulose is a linear homopolymer of d-glucose units linked by 1,4-β-d-glucosidic bonds, known to be semicrystalline and highly recalcitrant to enzymatic hydrolysis [1]. Hemicelluloses are mostly made up of pentoses (xylose and arabinose mainly), with smaller contributions of hexoses (mannose, glucose and galactose) and sugar acid heteropolymers [1]. Lignin is a phenolic polymer, which forms a tridimensional structural network surrounding the cellulose and the hemicellulose fractions [4]. Lignin has a complex structure and is very resistant to enzyme attack and degradation, thus representing one of the most important factors determining cell wall recalcitrance to hydrolysis [1, 3, 5].

Currently, lignocellulosic biomass conversion to fermentable sugars represents significant technical and economic challenges and its success depends mostly on the development of effective pretreatments and on less expensive enzymatic hydrolysis processes. The former are necessary to decrease the above-mentioned recalcitrance, facilitate the enzymatic hydrolysis to fermentable sugars and minimize the stress-tolerant microbial biocatalysts [6–10].

The crystallinity index (CI) has been used to interpret changes in cellulose structure after biological and physicochemical pretreatments. While some results obtained with lignocellulosic biomass demonstrate a progressive increase in the CI of the samples as a function of pretreatments [9, 11, 12], other studies reported [13] that the crystalline index of poplar cellulose remained almost unchanged during acid pretreatments. On the other hand, it has also been shown that the CI can significantly vary depending on the choice of the measurement method [14]. Moreover, as stated in Refs. [15, 16], the crystallinity index of biomass is also sensitive to change in composition, which is likely to occur during pretreatments, for example, removal of lignin and hemicelluloses. Hence, it is not a good index for characterizing the effect of pretreatments on the crystallinity of cellulose. Therefore, it is crucial to differentiate between the crystallinity of the biomass from that of the cellulose inside the biomass.

The CI of lignocellulose biomass samples, calculated from X-ray diffraction (XRD) or ^13^C solid-state nuclear magnetic resonance (ssNMR), usually reflects the total sample crystallinity. On the other hand, specific information about the native cellulose crystallinity within the biomass, i.e., the fraction of the ordered domains (usually referred to as crystalline) over the total amount of cellulose within the biomass structure is difficult to obtain. It happens because hemicellulose, lignin and disordered cellulose domain together contribute toward the amorphous phase signal of the sample [14]. Thus, the possibility of evaluating the CI of native cellulose in biomass with minor influence of the lignin and hemicellulose fractions is highly desirable. Indeed, this is particularly important for a better evaluation of the possible variations in the CI taking place as a consequence of pretreatments, when the hemicellulose and lignin fractions vary significantly.

In this respect, an important achievement was attained by Ragauskas and co-workers, who proposed a method to isolate the cellulose from the biomass. The effect of the isolation on the ultrastructure of the cellulose was studied using ^13^C CPMAS NMR [7], showing an apparent increase of cellulose crystallinity of only ~10% or less.

As reviewed in Refs. [7, 12], the influence of the CI on the recalcitrance of biomass was for a long time controversial, but the most recent results tend to indicate that the efficiency of pretreatments in reducing the recalcitrance is not clearly correlated with the decrease of the CI, with other parameters such as lignin/hemicelluloses contents, sample morphology and porosity, polymerization degree, lignin and hemicellulose distribution also being very, or even more, important [7, 17, 18]. In particular, as concluded in Ref. [7], improving the accessibility of the enzymes to the cellulose seems to be the main player in reducing the recalcitrance of the biomass, with the cellulose crystallinity being a second-order aspect.

Here, we propose an alternative method to obtain information on the cellulose CI directly within sugarcane bagasse samples, which had undergone chemical pretreatment (acid + alkaline) alone or followed by enzymatic hydrolysis using ssNMR. This was achieved using a spectral editing procedure that removed the lignin signals from the ssNMR spectrum, acquired using the recently proposed MultiCP pulse sequence that provides quantitative ^13^C NMR spectra [19] with good sensitivity. We also compared our results with XRD data using deconvolution and peak height methods, revealing that, despite the clear increase of the biomass crystallinity index as measured by XRD, there were no significant variations in the CI of cellulose inside the biomass induced by the chemical pretreatments undergone here. These findings definitely confirm that great care should be taken when associating the biomass crystallinity index as one of the main factors in circumscribing the rate of lignocellulosic biomass hydrolysis by enzymatic mixtures [7, 16]. The method has the drawback of requiring a lignin standard spectrum, but can be efficiently used to estimate the CI of the cellulose inside the intact biomass when analyzing changes in cellulose crystallinity in samples with similar origins.

## Results and discussion

### Spectral assignment of the ^13^C NMR spectra of sugarcane bagasse

**Fig. 1:**
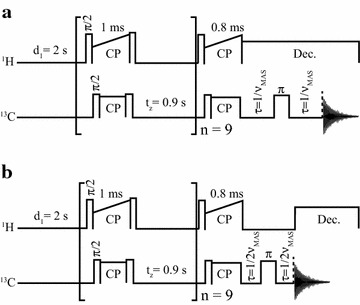
NMR methods used. **a** Sketch of the MultiCP pulse sequence used to obtain quantitative ^13^C spectra. **b** Sketch of the MultiCP with dipolar filter sequence used to acquire selective spectra from hemicellulose and lignin inside hydrated biomass samples.

The pulse sequences used in the NMR analysis are shown in Fig. 1. The MultiCP pulse sequence (Fig. 1a) provides quantitative ^13^C NMR spectra [19] with good sensitivity, thus enabling curves with good signal-to-noise ratio in reduced spans of time. Using this process, the changes in the sugarcane bagasse sample could be followed after each step of the pretreatment and enzymatic hydrolysis.

**Fig. 2:**
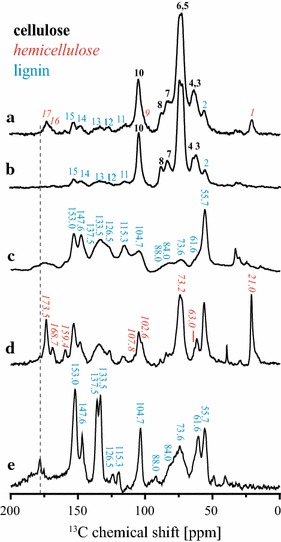
Examples of NMR spectra acquired. MultiCP spectra with or without dipolar filter obtained in sugarcane bagasse before and after pretreatments: (*a*) spectrum of hydrated untreated bagasse acquired with Multi-CP; (*b*) spectrum of hydrated bagasse after H_2_SO_4_ pretreatment acquired with Multi-CP; (*c*) spectrum of lyophilized supernatant from the pretreatment with 1% H_2_SO_4_ + 0.25% NaOH acquired with Multi-CP; (*d*) spectrum of hydrated untreated bagasse acquired with dipolar filtered Multi-CP; (*e*) spectrum of hydrated bagasse after H_2_SO_4_ pretreatment acquired with dipolar filtered Multi-CP.

As already stated, the main aim of this work is to evaluate the CI of the cellulose fraction within the plant biomass using ssNMR. Prior to that, however, it is important to have a reliable identification of the NMR lines resulting from each of the biomass components. Figure 2a shows the MultiCP spectrum of untreated bagasse. The peak pattern is the same as that presented in Ref. [4], however, with some differences in the relative line intensities due to the use of the MultiCP. Based on the published data [4, 20–24], lines 1–17 are similarly assigned, with different colors (black, red or blue) indicating the most evident lines of each main component of the biomass (cellulose, hemicellulose or lignin, respectively). Nevertheless, due to the chemical complexity of the biomass material, a strong line overlap is expected and observed. This is evident in Fig. 2b, which shows the Multi-CP spectrum of a sugarcane bagasse sample pretreated with 1% H_2_SO_4._ Besides the absence of lines 9, 16 and 17 (due to removal of the hemicellulose fraction), it is possible to observe an increase in the spectral resolution in the 50–70 ppm region, which suggests the removal of other hemicellulose lines that overlap with the cellulose signals in that region. In addition, there is also an overlap with lignin signals, as shown in Fig. 2c, where the spectrum of the lyophilized hydrolysate (supernatant) resulting from the two-step pretreatment of sugarcane bagasse (first with 1% H_2_SO_4_ and then with 0.25% NaOH) is presented. As previously discussed [4], this spectrum is only composed of lignin signals, which are distributed throughout the whole spectral region. Signals labeled as 2 (55.7 ppm), 11 (115.3 ppm), 12 (126.5 ppm), 13 (133.5 and 137.5 ppm), 14 (147.6 ppm) and 15 (153.0 ppm) in Fig. 2a are exclusively due to lignin [4, 25, 26], while other lignin signals coincide with cellulose peaks, for instance, at 61.6, 73.6, 84.0, 88.0 and 104.7 ppm [25, 26].

Although most of the lignin signals in Fig. 2c can be directly assigned to lignin, there are peaks, particularly those in the 50–120 ppm region, which need to be certified as not having a hemicellulose contribution. Thus, for a more precise evaluation of the cellulose CI, it is important to confirm that practically all the hemicellulose signals vanish with 1% H_2_SO_4_ treatment. To do so, the method described by Komatsu and Kikuchi [25–27] was applied. It consists of acquiring a dipolar filtered ^13^C NMR spectrum from a hydrated lignocellulose sample. Then, the dipolar filter removes the signal arising from rigid segments by applying a rotor synchronized dipolar dephasing pulse sequence, as shown in Fig. 1b. While the molecular motions in the cellulose chains are not altered by the hydration, this hydration led to an increase in the rate of molecular motions on the hemicellulose and lignin segments. Therefore, the acquisition of the dipolar filtered spectrum of the hydrated biomass results in a dipolar edited spectrum, which contains only hemicellulose and lignin signals. This experiment was carried out by hydrating the untreated sugarcane bagasse sample with Milli-Q water and then applying the MultiCP followed by dipolar filter experiment (Fig. 1b), which resulted in the spectrum shown in Fig. 2d. The suppression of the cellulose signals is evident, as only signals from lignin and hemicellulose remain (indicated in Fig. 2d) [25–27]. Note that the lignin signals appear at the same chemical shifts as at the spectra of Fig. 2c, but due to the application of the dipolar filter and the sample hydration, the relative intensities and widths are not directly comparable. Despite that, by exclusion, the hemicellulose signals can be identified, including lines 1 and 17, which are clearly visible in the untreated bagasse spectra, and also signals at 63, 73.2, 102.6, 107.8 and 159.4 ppm, which were hidden in the spectrum of the untreated sugarcane bagasse [4].

Figure 2e shows the dipolar filtered MultiCP spectrum of hydrated sugarcane bagasse after acid (H_2_SO_4_) pretreatment. The removal of all hemicelluloses lines is clearly observed, showing the effectiveness of the acid treatment in removing the hemicellulose components Note that the chemical shifts of the two pronounced lines in the 170–200 ppm do not have the same values as the hemicelluloses peak shown in Fig. 2a, d (dashed vertical line).

### Spectral editing for evaluating the cellulose CI in sugarcane bagasse

Information on CI can be obtained using ssNMR by the deconvolution of lines at 84 and 88 ppm, which are assigned to the C4 carbon of amorphous and crystalline cellulose, respectively. However, as shown in Fig. 2c, signals from lignin also contribute to the spectrum in this region. The positive point is that the contribution from hemicellulose signals is minor and can be suppressed by treating the bagasse samples with H_2_SO_4_. Hence, the general idea behind the calculation of the CI of the native cellulose of biomass is to obtain quantitative ^13^C NMR spectra only associated with the cellulose carbons, i.e., without contribution from lignin. This can be accomplished by subtracting the pure lignin Multi-CP spectrum from that of biomass scaled by a factor that can be determined by matching the amplitudes of the common signals at both spectra, for instance, lines 12 and 13 in Fig. 2a. The pure lignin spectrum could be, in principle, obtained from a standard sample, but because the lignin composition varies with the origin and the type of biomass, it is more accurate to use a lignin sample extracted from the same biomass. Fortunately, the spectrum of the lyophilized supernatant obtained after the two-step pretreatment with 1% H_2_SO_4_ and 0.25% NaOH only contains lignin signals [4], as shown in Fig. 2c, and can be used as a pure lignin spectrum.

**Fig. 3:**
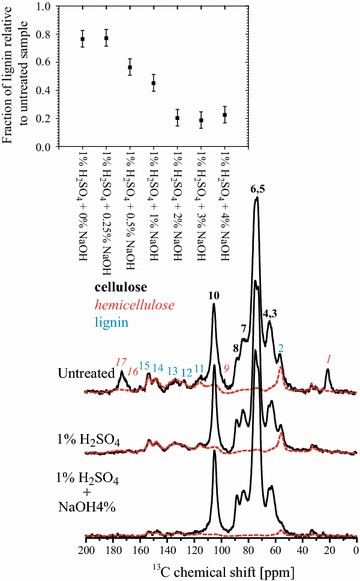
Effects of pretreatments in the NMR spectra. MultiCP spectra with chemical shift assignments obtained in sugarcane bagasse under three pretreatment conditions (untreated, 1% H_2_SO_4_ and 1% H_2_SO_4_ + 4% NaOH). The *red dotted lines* represent the MultiCP spectrum of the lyophilized supernatant resulting from a pretreatment with 1% H_2_SO_4_ + 0.25% NaOH. The *inset* shows the lignin fraction in each sample with respect to the untreated bagasse, obtained from the scaling factor that matches the lignin signals of the supernatant with those from the solid pretreated biomass.

Figure 3 shows MultiCP spectra of sugarcane bagasse before and after undergoing pretreatments with 1% H_2_SO_4_ and 1% H_2_SO_4_ + 4% NaOH, together with the respective scaled spectrum of the lyophilized supernatant. Note that the scaling factor provides the relative quantity of lignin in each treated sample. Thus, the inset of the figure shows the obtained relative fraction of lignin on the samples treated with 1% H_2_SO_4_ and NaOH at different concentrations, in terms of the untreated bagasse. A good agreement between the lignin signals in the lyophilized supernatant and those in the pretreatment samples can be noticed. Nevertheless, one should state that for biomasses of the distinct kinds, the lignin standard should be different and this would be a drawback of the method. Indeed, at least for grasses, the (1%) H_2_SO_4_ + (0.25%) NaOH treatment at the temperature and time conditions described in the experimental section would be a suitable method for extracting lignin that could be used as a standard in our subtraction method for evaluating CI.

The profile of lignin removal (inset) is quite comparable with that previously obtained by high-performance liquid chromatography (HPLC) [4]. The lignin extraction was mostly due to the alkaline pretreatment at NaOH concentrations of 2% or higher, and more than 80% of the lignin in the untreated sample was removed, which is in full agreement with the previous work [4].

**Fig. 4:**
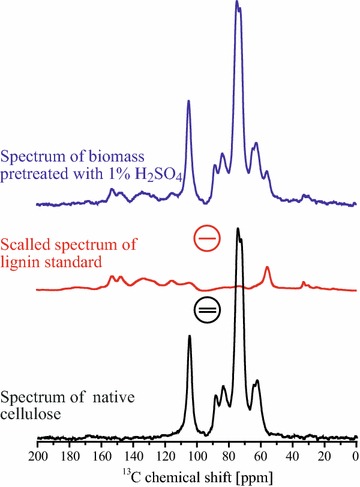
NMR spectral subtraction. Illustration of the spectral subtraction of lignin signals from sugarcane bagasse spectra to obtain a spectrum of native cellulose within the biomass.

**Fig. 5:**
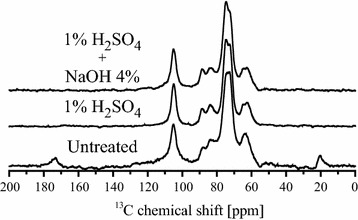
Pure cellulose NMR spectra. MultiCP spectra after the removal of lignin signals for untreated bagasse and bagasse samples pretreated with 1% H_2_SO_4_ and 1% H_2_SO_4_ + 4% NaOH.

As already stated, the spectrum in Fig. 2c retains all the features from the lignin signal in the sample, it is possible to remove their contribution from the sugarcane bagasse spectrum by simple spectral subtraction. In general, the spectrum resulting from the subtraction includes contributions from cellulose and hemicellulose. But after the 1% H_2_SO_4_ treatment, as in the example shown in Fig. 4, the resulting spectrum accounts just for the cellulose signals; see Fig. 5 after the 1% H_2_SO_4_ treatment and after 1% H_2_SO_4_ + 4% NaOH treatment. Therefore, based on the deconvolution of the C4 carbon signals [9, 11, 14], it is possible to obtain the CI of the native cellulose in sugarcane bagasse. Note that in the untreated sugarcane bagasse sample in Fig. 5, the hemicellulose signals are still present, but since they do not contribute significantly in the 80–88 ppm range, the CI of cellulose can still be roughly estimated from the subtraction approach.

### Evaluating the cellulose CI in sugarcane bagasse undergoing chemical pretreatments

**Fig. 6:**
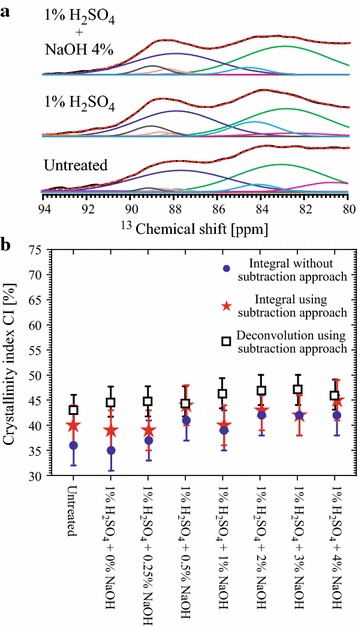
Effect of pretreatments in NMR crystallinity indexes. **a** MultiCP spectra amplified in the 80–94 ppm region, with the respective Gaussians/Lorentzians deconvolutions [14] for untreated bagasse and bagasse pretreated with 1% H_2_SO_4_ and with 1% H_2_SO_4_ + 4% NaOH; **b** CI values of sugarcane bagasse after each pretreatment step obtained according to the three procedures indicated.

After carrying out the spectral subtraction procedure, the spectral regions from 80 to 86 and 86 to 94 ppm were integrated to evaluate the intensity of the lines associated with ordered and disordered cellulose phases, respectively (*12*). To evaluate the influence of lignin removal in the cellulose CI obtained by NMR, the same procedure was also carried out using the spectra without subtraction. The CI results are shown in Fig. 6b. The CI values obtained without using the spectral subtraction approach (stars in Fig. 6b) show a slight, but progressive increase upon alkaline pretreatment due to lignin removal. Nevertheless, the CI values obtained using the spectral subtraction (circles in Fig. 6b) remained mostly constant upon pretreatment. Therefore, within the experimental uncertainties, both calculations point to a constancy of the cellulose CI inside the biomass. Thus, despite that the subtraction method would provide more precise evaluation of the CI, the ^13^C-solid-state Multi-CP NMR also provides a fair estimation of the CI of cellulose inside the biomass. This is justified by the presence of only small lignin signals in the C4 spectral region even for the intact biomass.

The possibility of obtaining a virtually pure cellulose spectrum allows using a deconvolution-based approach to evaluate the CI of cellulose. To do so, based on Refs. [9, 11, 14], line 7 in Fig. 3, which is assigned to the C4 carbon of disordered cellulose, was fitted using Gaussian lines at 83.1 and 84.2 ppm, due to accessible fibril surfaces, and at 81.4 ppm, due to inaccessible fibril surfaces. In the region of line 8 in Fig. 3 (C4 carbon of ordered cellulose), a Gaussian line at 88.5 ppm was used to account for paracrystalline cellulose, and three Lorentzian lines were used to account for crystalline cellulose: at 87.9 ppm for the β phase, 88.7 ppm for the α + β phase and 89 ppm for the α phase. Thus, the CI is calculated by the ratio between the integrated intensities of the lines associated with the ordered cellulose and the total area of the lines assigned to the C4 carbon (ordered + disordered cellulose) [9, 11, 14]. Figure 6a shows an example of the deconvolution of the C4 carbon signal using Gaussian and Lorentzian lines for untreated bagasse samples and samples pretreated with 1% H_2_SO_4_ and 1% H_2_SO_4_ + 4% NaOH.

The CIs calculated by the deconvolution of the subtracted spectra are shown Fig. 6b for the untreated sample, as well as for samples pretreated with H_2_SO_4_ and NaOH at varying concentrations. The calculation of the CI values were done using CI values calculated for all the samples and are nearly constant (CI ~45%), suggesting that the cellulose crystallinity inside the biomass is not considerably altered by the pretreatments.

**Fig. 7:**
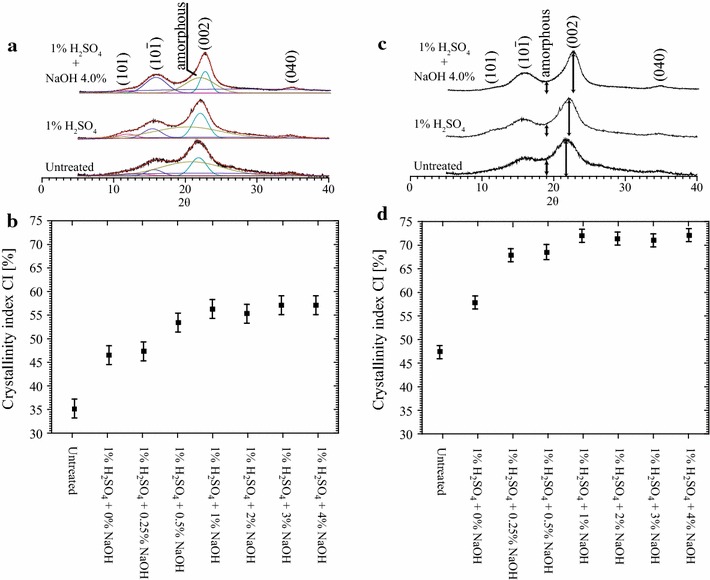
Effect of pretreatments in X-ray crystallinity indexes. **a** X-ray diffractograms of untreated bagasse and bagasse pretreated with 1% H_2_SO_4_ and with 1% H_2_SO_4_ + 4% NaOH, analyzed by the deconvolution XRD method; **b** CI values obtained by the deconvolution method after each pretreatment step; **c** XRD diffractograms of untreated bagasse and bagasse pretreated with 1% H_2_SO_4_ and with 1% H_2_SO_4_ + 4% NaOH, analyzed by the peak height XRD method; **d** CI values obtained by the peak height method after each pretreatment step.

To provide a comparison between the CI values calculated using the aforementioned ssNMR analysis with those obtained using the standard X-ray diffraction methods, XRD diffractograms are presented in Fig. 7a, c. The diffractograms in Fig. 7 represent the same set of samples analyzed by ssNMR and presented in Fig. 6a. The sample CI values from XRD data were calculated using the two most common procedures used for this purpose: the deconvolution of the XRD diffractogram and the peak height method [9, 11, 14]. In the first case, the XRD diffractogram is deconvoluted, so that the full intensities associated with the crystalline reflections of cellulose ($101,10\bar{1}, 002,040$) and the amorphous halo are found (Fig. 7a). The CI value is then calculated from the ratio between the intensities of the crystalline reflections of cellulose and the total area of the diffractogram. The CI results obtained by this method are presented in Fig. 7b.

In the peak height method, the CI value is calculated from the height ratio between the intensity of the crystalline peak (*I*_002_ − *I*_AM_) and total intensity of the peak (*I*_002_), considering that *I*_AM_ is the intensity at the dip between the 002 and the $10\bar{1}$ peaks, as shown in Fig. 7c. The CI values obtained by the peak height method are shown in Figs. 7d. Previous reports indicated that, for pure cellulose samples, the CI values obtained by deconvolution of the XRD and by ssNMR tend to agree, while the XRD method by peak height tends to overestimate the crystalline fraction [14, 28]. This is also observed in our data for samples treated under different conditions. While the CI values calculated by the peak height method vary from 45 to 75%, the CIs calculated by the deconvolution method vary from 35 to 55%.

It is worth mentioning that, because the amorphous region observed in XRD refers to the total amorphous fraction of the sample and does not distinguish the source of the amorphous material (amorphous cellulose, hemicellulose or lignin), the CI obtained by XRD refers to the whole sample and not only to the cellulose fractions. Therefore, when hemicellulose or lignin is removed from the sample, the CI tends to increase, due to a proportional reduction in the amorphous halo of the XRD diffractogram. This is clearly observed in Fig. 7b, d, where CI increased from ~35% for the untreated sample to ~47% in the samples treated only with H_2_SO_4_. Considering the ssNMR results shown in Fig. 6, this CI increase is mostly associated with the removal of hemicellulose. A similar conclusion can be drawn in the case of NaOH pretreatment, where the increase in the CI values as a function of the NaOH concentration is mostly associated with lignin removal. Besides the ssNMR results in Fig. 6, this is also shown by the constant CI values in samples treated with NaOH concentrations higher than 1% (Fig. 7b, d). As shown in Ref. [4] and Fig. 3, lignin removal at these concentrations is similar. Note that ^13^C ssNMR probes as crystalline all domain that present local conformational order, while in XRD only those domains presenting long range order are seen as crystalline. Therefore, the CI seen by ^13^C ssNMR and XRD may not have exactly the same value.

**Fig. 8:**
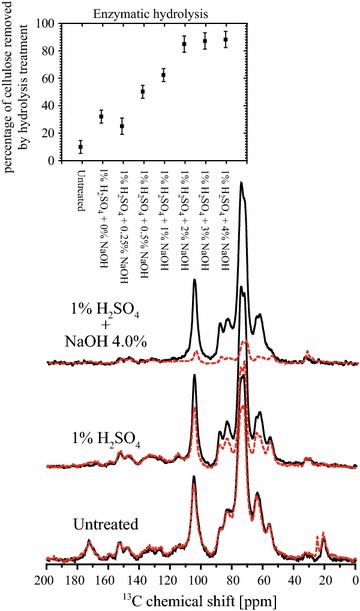
Effects of pretreatments followed by enzymatic hydrolysis in the NMR spectra. MultiCP spectra of sugarcane bagasse samples before and after chemical pretreatments (*solid black lines*) compared to samples that were also submitted to enzymatic hydrolysis after the chemical pretreatments (*dashed red line*). Curves were normalized considering the lignin signals at 120–160 ppm regions. The *inset* shows the percentage of cellulose removed by the enzymatic treatment obtained from the normalization scaling factors.

The quantitative MultiCP ssNMR spectra were also used to estimate the amount of cellulose removed by enzymatic hydrolysis. This is achieved by comparing the spectra of the non-hydrolyzed samples with those of the remaining solid fraction after enzymatic hydrolysis [4]. Considering that the enzymatic treatment does not cause changes in the lignin fraction, the lignin signals are not expected to be altered by enzymatic hydrolysis. Hence, the lignin signals in the spectra of the remaining solid fraction after hydrolysis can be scaled to match those in the non-hydrolyzed samples, as shown in Fig. 8. By doing so, the ratio between the intensities of line 10 at 104.7 ppm, assigned to C1 carbon of cellulose, is a measure of the relative amount of digested cellulose.

Figure 8 shows the obtained percentages of the digested cellulose in samples submitted to different pretreatment conditions. As it can be observed, cellulose digestion reaches values with excess of 90% for the samples pretreated with 1% H_2_SO_4_ and 2% NaOH or higher. These results follow the same trends that were previously observed for the enzymatic yields in sugarcane bagasse under similar pretreatments and quantified by HPLC [4, 29].

**Fig. 9:**
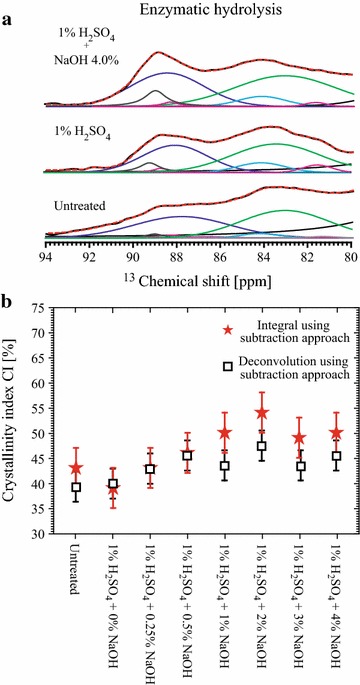
Effect of pretreatments in NMR crystallinity indexes. **a** MultiCP spectra amplified in the 80–94 ppm region, with the respective Gaussians/Lorentzians deconvolutions obtained in bagasse samples pretreated with 1% H_2_SO_4_ and with 1% H_2_SO_4_ + 4% NaOH and then submitted to enzymatic hydrolysis; **b** CI values of the remaining native cellulose after the enzymatic hydrolysis as a function of the chemical pretreatments. The two procedures were used in the evaluation of the CIs.

Cellulose CI in the remaining solid fraction after the enzymatic hydrolysis was also evaluated by ssNMR, using the subtraction procedure. The C4 signal deconvolutions for untreated samples, samples pretreated with 1% H_2_SO_4_ or 1% H_2_SO_4_ + 4% NaOH are shown in Fig. 9a. The cellulose CIs of all the analyzed samples calculated using simple integration [14] and deconvolution [9, 11] are shown in Fig. 9b. It can be observed that the CI values show only about 10% increase upon enzymatic hydrolysis, which indicates that the particular enzyme cocktail used here does not have strong preference to digest crystalline or amorphous cellulose. However, one should stress that the cellulose fraction in each ssNMR spectrum refers to the remaining cellulose in the samples, i.e., the fraction which was not consumed by the enzymes. For instance, in the samples pretreated with NaOH at concentrations higher than 2%, the remaining cellulose fraction represents less than 10% of the initial quantity of cellulose. Besides, one should not disregard the possibility of hornification upon drying [30–35], which cannot be ruled out as a cause of the not significant variation in the cellulose CI upon the pretreatments. This can be investigated futher using the method presented in this article.

The idea that the enzymes used here do not show a clear preference for crystalline or amorphous cellulose should be taken with a pinch of caution [7]. More efforts to confirm this finding and also to evaluate the effects of other enzymatic mixtures on the plant cell walls crystallinity are in progress.

## Conclusions

The effects of consecutive acid/alkaline pretreatments and enzymatic hydrolysis on sugarcane bagasse were studied herein using quantitative Multi-CP ^13^C ssNMR spectroscopy. The removal of hemicellulose and lignin due to the pretreatments was selectively evaluated, as well as the crystalline index of cellulose inside the biomass, by spectral editing. Besides quantifying the relative amount of removed lignin, the results revealed that the acid/alkaline pretreatment does not result in important modifications in the crystalline index of cellulose. The relative amount of cellulose removed by enzymatic hydrolysis was also evaluated by ssNMR in samples previously treated under different acid/alkaline concentrations, and the behavior observed was in a good agreement with the data obtained by HPLC from the same samples. Furthermore, the evaluation of the CI of cellulose in the remaining solid fraction after the enzymatic hydrolysis suggests that the particular commercial enzyme cocktail used in the presented work does not show a clear preference for digesting amorphous or crystalline cellulose fractions within the sugarcane bagasse. These findings support other literature work in the claim that crystallinity is not a primary factor that defines plant biomass recalcitrance. The presented method may be useful in evaluating the action of different pretreatments and novel enzymatic mixtures for plant cell wall digestion upon cellulose ultrastructure in lignocellulosic biomasses.

## Methods

Nuclear magnetic resonance NMR experiments were performed using a Bruker Avance 400 spectrometer, equipped with a Bruker 4-mm MAS double-resonance probe head, at ^13^C and ^1^H frequencies of 100.5 and 400.0 MHz, respectively. The spinning frequencies (at 14 kHz) were controlled by a pneumatic system that ensures a rotation stability higher than ~1 Hz. Typical *π*/2 pulse lengths of 4 and 3.5 µs were applied for ^13^C and ^1^H, respectively. Proton decoupling field strength of γB1/2*π*  = 70 kHz was used. ^13^C quantitative spectra were measured by using the multiple-CP (MultiCP) excitation method described by Johnson and Schmidt-Rohr [19]. A total of nine CP blocks were implemented with 1 ms and RF amplitude increment (90–100%), while the last CP before acquisition was executed with 0.8 ms and the same amplitude increment. The recycle delay was 2 s and the duration of the repolarization period *t*_z_ was 0.9 s [19]. A Multi-CP excitation followed by a DIPSHIFT type of dipolar filter with duration $[ 1/ 2\nu_{\text{MAS}} {-} 1 80^\circ {-} 1/ 2\nu_{\text{MAS}} ]$ was applied to a hydrated sample, to obtain a ^13^C spectra selective for the hemicellulose and lignin signals of sugarcane bagasse [27].

X-ray diffraction data were collected with a Rigaku Rotaflex diffractometer model RU200B (Tokyo, Japan) using monochromatic CuKa radiation (1.54 Å). The goniometer scanned a 2*θ* range between 5° and 65° at a 2°/min scanning rate. Samples were knife milled prior to analysis until they were put through a 2 mm sieve. The CI for all the samples was calculated according to the XDR deconvolution and peak height methods [4, 14]. The deconvolution of the XRD diffractograms was performed using four Gaussian peaks to account for the crystalline diffractions of cellulose ($10 1,{ 1}0{\bar{\text{1}}},00 2 {\text{ and }}0 40$) and one Gaussian to the amorphous fraction of the sample. The crystallinity index was calculated as the ratio between the sum of the areas of all the crystalline peaks and the total area of the spectrum. The peak height method considers the total intensity of the peak (*I*_200_) and the intensity due to the crystalline peak only, which is obtained by subtraction of the intensity of the amorphous dip (*I*_200_ − *I*_AM_). The CI in the height method is thus obtained as the ratio (*I*_200_ − *I*_AM_)/*I*_200_. The background subtraction was carried out considering the spectrum obtained for an empty sample holder in the same 2*θ* range.

Pretreatments on sugarcane bagasse samples were carried out following a two-step procedure. In the first step sugarcane bagasse was hydrolyzed with diluted H_2_SO_4_ (1% v/v in water) for 40 min at 120°C, where the pressure was kept at 1.05 bar and a 1:10 solid to liquid ratio (grams of bagasse/ml of solution) was used. In the second step, NaOH solutions were used with increasing concentrations (0.25, 0.5, 1.0, 2.0, 3.0 or 4.0% w/v) at 120°C for 40 min. More details can be obtained from our previous article [4].

## Abbreviations

CI: crystallinity index
HPLC: high-performance liquid chromatography
Multi-CP: cross polarization by multiple contact periods
ssNMR: solid-state nuclear magnetic resonance
XRD: X-ray diffraction
